# National Institutes of Health funding for venous thromboembolism research

**DOI:** 10.1016/j.jtha.2026.01.020

**Published:** 2026-02-13

**Authors:** Ryan A. Coute, Jake Toy, Kameshwari Soundararajan, Benjamin von Schweinitz, Patrick J. Siler, Ryan C. Godwin, Ryan L. Melvin

**Affiliations:** 1 Department of Emergency Medicine, University of Alabama at Birmingham Heersink School of Medicine, Birmingham, Alabama, USA; 2 Los Angeles County Emergency Medical Services Agency, Santa Fe Springs, California, USA; 3 Department of Emergency Medicine, Harbor-University of California Los Angeles Medical Center, The Lundquist Institute, Torrance, California, USA; 4 Department of Emergency Medicine, David Geffen School of Medicine at University of California Los Angeles, Los Angeles, California, USA; 5 Department of Anesthesiology and Perioperative Medicine, University of Alabama at Birmingham Heersink School of Medicine, Birmingham, Alabama, USA; 6 Department of Radiology, University of Alabama at Birmingham Heersink School of Medicine, Birmingham, Alabama, USA

**Keywords:** National Institutes of Health, research funding, venous thromboembolism

## Abstract

**Background:**

Venous thromboembolism (VTE) is associated with approximately 100 000 deaths annually in the United States (U.S.). Progress in the prevention, diagnosis, treatment, and recovery from VTE depends on research funding. The National Institutes of Health (NIH), the world’s largest funder of biomedical research, does not currently report VTE-specific funding in its annual Categorical Spending Report.

**Objectives:**

This study aimed to provide a descriptive analysis of NIH funding for VTE research over the past decade.

**Methods:**

We conducted a search of the NIH Research Portfolio Online Reporting Tools Expenditures and Results database from 2015 to 2024 using a string of VTE-related search terms. Grants were categorized as VTE research (yes/no) using a large language model prompted with predefined classification criteria. We tabulated annual NIH funding amounts, the number of VTE-related grants, and the number of unique principal investigators. For 2023, VTE research investment was compared with that for heart disease and stroke, the leading causes of vascular mortality in the U.S.

**Results:**

The search yielded 2130 grants with complete data, of which 1114 were classified as VTE research. When excluding renewal awards, 490 unique VTE grants were identified. Total inflation-adjusted NIH funding for VTE research was $42 million in 2015, peaked at $73 million in 2021, and totaled $67.1 million in 2024. In 2023, NIH funding per annual deaths was $2765 for heart disease, $2724 for stroke, and $639 for VTE.

**Conclusion:**

NIH investment in VTE research has increased over the past decade, but remains disproportionately low relative to other major causes of vascular mortality in the U.S.

## 1 | INTRODUCTION

Venous thromboembolism (VTE) is a leading cause of mortality in the United States (U.S.), behind heart disease and stroke [1]. Although there is no specific VTE surveillance system in the U.S., the Centers for Disease Control and Prevention (CDC) estimates 100 000 VTE-related deaths annually [2]. VTE, which includes deep vein thrombosis and pulmonary embolism, occurs more frequently in older adults and in several high-risk groups, including individuals of Black race and those with sickle cell trait, factor V Leiden mutation, and obesity [3]. Research funding is critical to reducing the burden of VTE by improving preventative measures, diagnosis, treatment, and recovery. Limited data are currently available to understand investment in VTE research in the U.S. and the trend in funding over time.

The National Institutes of Health (NIH) is the world’s largest funder of biomedical research [4]. Each year, the NIH reports funding estimates for more than 350 disease categories in its Categorical Spending Report [5]. This report provides disease-specific funding information, including total funding, funding trends, and the number of grants. VTE is not included as a category, and funding details for VTE research are not available in the current NIH reporting [5]. Therefore, NIH investment in VTE research and its trend over time are largely unknown.

To address this knowledge gap, we conducted a descriptive analysis of NIH funding for VTE research over the past decade. The overall goals were to understand trends in funding over time and to compare NIH investment in VTE research with that for other leading causes of vascular morbidity and mortality.

## 2 | METHODS

### 2.1 | NIH funding for VTE research

A search of the NIH Research Portfolio Online Reporting Tools Expenditures and Results (RePORTER) database [6] was performed for the years 2015 to 2024 using the following string of search terms: “Venous thromboembolism,” “Pulmonary thromboembolism,” “Pulmonary embolism,” “Deep vein thrombosis,” “Deep venous thromboses,” “Venous thrombosis,” “Venous thromboses,” and “Thromboembolism.” All grants from non-NIH funding sources were excluded, including those from the Food and Drug Administration, the Department of Veterans Affairs, and the Agency for Healthcare Research and Quality. We also excluded grants with missing project abstracts, which are necessary for grant categorization.

The search results were directly exported to a Microsoft Excel spreadsheet (Microsoft Corporation). The available data included the project abstract, funding mechanism, funding institute, contact principal investigator, award type, grant mechanism, and total annual funding. The project abstract for each grant was individually reviewed by a large language model (LLM) and categorized as VTE research (yes/no) based on predetermined inclusion and exclusion criteria (Table 1).

### 2.2 | LLM grant categorization

The categorization process involved the following steps: first, the research data file was read using the Python (version 3.11.4) Pandas (2.3.1) libraries, and the column containing the research abstracts (unstructured text data) was identified as the LLM input. Next, a set of predefined categories and their descriptions from Table 1 were used as input to create a so-called “prompt” for the LLM.

Next, the abstracts and categories with descriptions were processed using the GPT-4o mini (OpenAI) LLM, with a prompt instructing it to apply 1 of the provided category labels to the abstract and its label description. The LLM’s temperature parameter was set to 0 to minimize randomness. Other LLM parameters (Table 2) were based on a previously published framework [7].

Classification was performed using a zero-shot learning approach, in which each abstract was classified independently without any labeled training examples [7]. That is, the LLM received exactly 1 abstract at a time, with no information about any other abstracts. Upon completion of the LLM-based categorization of all included abstracts, the output was generated as a new Excel file that included the original dataset, the LLM-assigned category for each abstract, and the LLM-generated chain-of-thought rationale to support each classification decision. In the chain-of-thought framework, the LLM is instructed to provide a rationale before the final classification decision, so that the rationale is used in decision-making rather than serving as a justification for the decision [7].

### 2.3 | Statistical analysis

The data were analyzed descriptively. The primary outcome was inflation-adjusted annual funding for VTE research, expressed in millions of dollars. Inflation adjustment was performed for the funding years 2015 to 2023 using the Consumer Price Index Inflation Calculator provided by the U.S. Bureau of Labor Statistics [8].

Secondary outcomes included the number of principal investigators (defined as the contact principal investigator), the number of grants funded per year, the number of R01 grants, and the common funding institutes. As measures of the early-career investigator pipeline, the number of mentored K awards and the total K award funding were captured by year. Mentored K awards were defined as K01, K08, K23, or K99 mechanisms, based on similar analyses [9,10]. NIH investment in VTE research (defined as dollars invested per annual death) was also calculated. This investment was compared with the 2 most common causes of vascular mortality in the U.S., heart disease and stroke, based on CDC data [1]. NIH funding for heart disease and stroke research was obtained from the NIH Categorical Spending Report for 2023 [5]. Total deaths due to heart disease and stroke were extracted from the CDC National Vital Statistics Reports for 2023 [1]. Deaths due to VTE are not included in National Vital Statistics reporting but are estimated by the CDC to be approximately 100 000 annually [2].

Two independent human reviewers manually reviewed and categorized the individual grants for funding year 2015 (*n* = 163) based on information provided in the grant abstracts, using the same inclusion/exclusion criteria (Table 1) as those provided to the LLM as a “prompt.” Interrater reliability between the 2 human reviewers was assessed using Cohen’s kappa. Interrater reliability between the LLM categorization and the 2 independent human reviewers for the funding year 2015 was estimated using Fleiss’ kappa.

### 2.4 | Code availability

For those wishing to reproduce this process, the code used for classification is freely available on GitHub at https://github.com/UABPeriopAI/label_maker, and the computational steps are described in greater detail in our technical preprint [11].

## 3 | RESULTS

**FIGURE 1:**
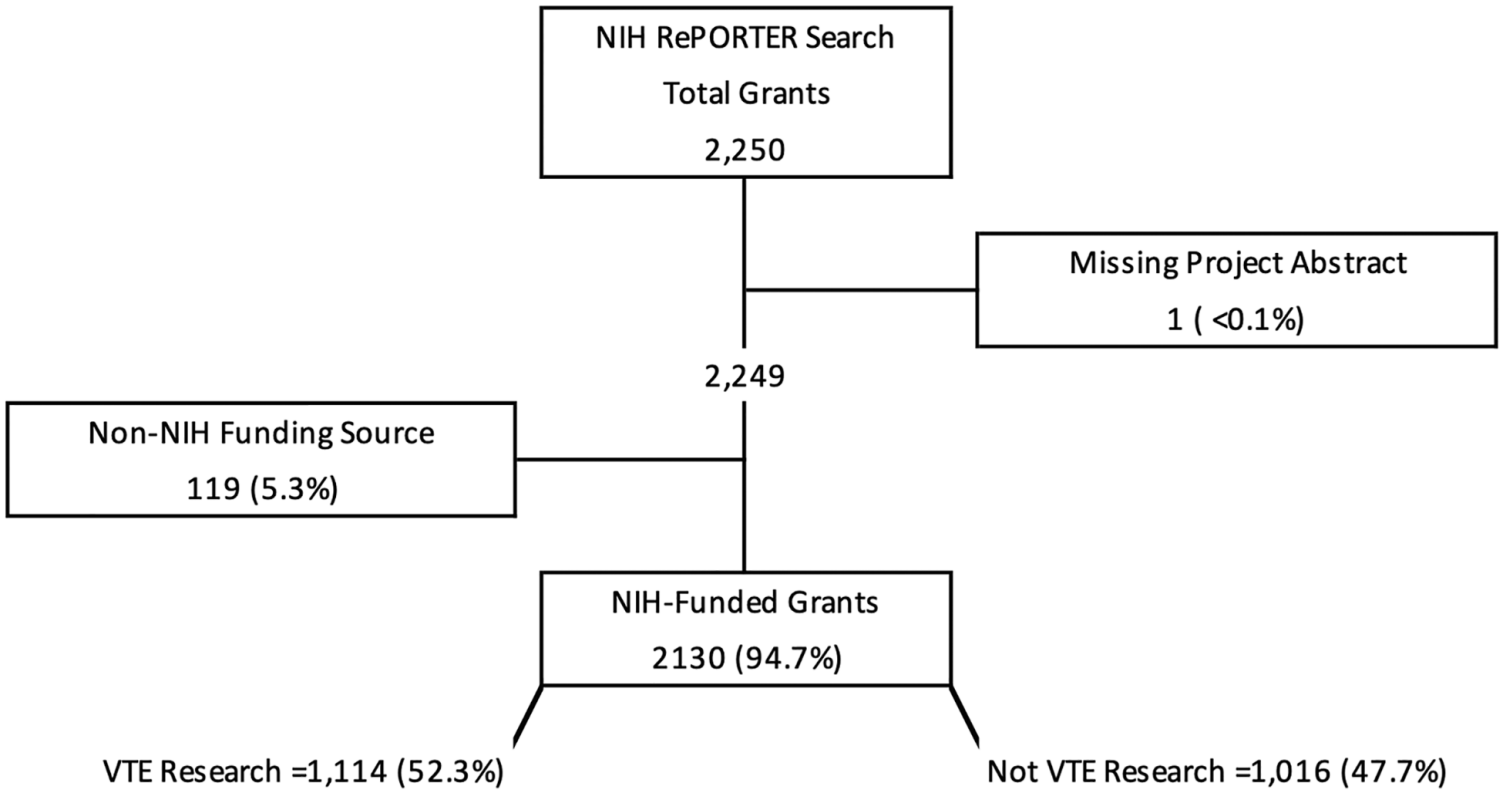
Flow diagram of grants extracted from the National Institutes of Health (NIH) Research Portfolio Online Reporting Tools Expenditures and Results (RePORTER) database for funding years 2015 to 2024. VTE, venous thromboembolism.

The NIH RePORTER database search yielded 2250 grants from a total of 797 212 awarded over the time period. One grant was excluded due to a missing abstract, and 119 were excluded because their funding sources were non-NIH (Food and Drug Administration, Department of Veterans Affairs, or Agency for Healthcare Research and Quality). Of the remaining 2130 grants, 1114 (52.3%) met study inclusion criteria and were classified by the LLM as VTE research (Figure 1). Fleiss’ kappa for interrater reliability comparing grant categorization for the financial year (FY) 2015 between the LLM and 2 independent human reviewers was 0.71 (95% CI, 0.62–0.80), indicating good interrater agreement. Cohen’s kappa for interrater reliability between the 2 independent human reviewers for FY2015 was 0.95 (95% CI, 0.89–0.99).

**FIGURE 2:**
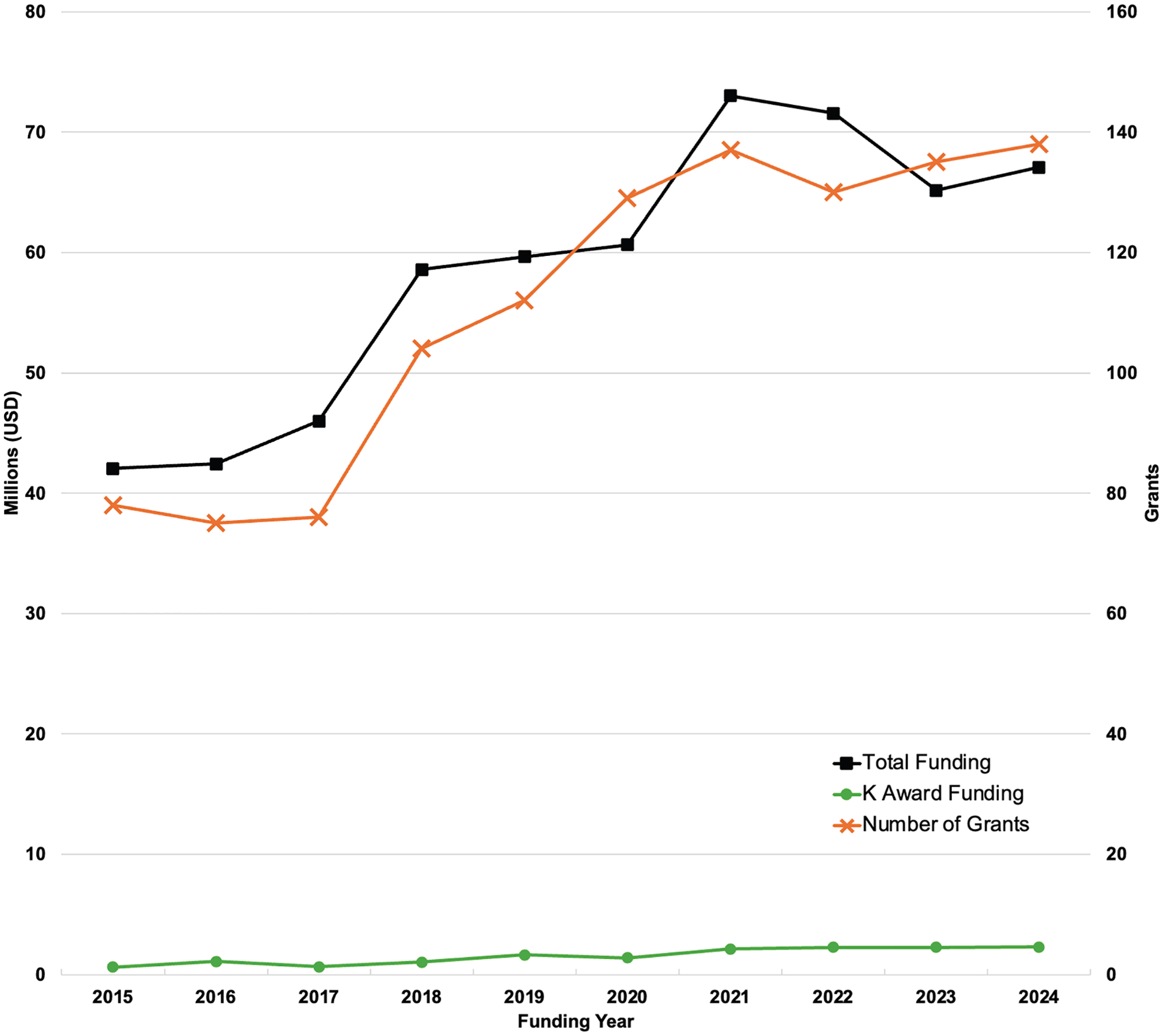
National Institutes of Health funding for venous thromboembolism research. Note: data for funding years 2015 to 2023 are adjusted for inflation. Mentored K Awards included K01, K08, K23, and K99.

The total funding for VTE research over the 10-year study period was $506.4 million, awarded to 341 principal investigators. Funding for the index year of 2015 was $31.6 million (inflation-adjusted: $42 million) and increased to $67.1 million in 2024, representing a 60% increase over the study period (Figure 2). The maximum inflation-adjusted funding was in 2021, at $73 million. The R01 mechanism accounted for 460 (41.3%) of the total awards, whereas mentored K awards accounted for 83 (7.5%; Table 3). Inflation-adjusted funding for mentored K awards increased more than 3-fold over the study period, from $623 000 in 2015 to $2.3 million in 2024.

In FY2023, the NIH allocated approximately $63.9 million to VTE research, representing 0.1% of its total grant funding of $44.9 billion [12]. By comparison, heart disease and stroke received 4.2% and 1.0% of the total funding, respectively [5]. When adjusted for the number of annual deaths, the NIH investment amounted to approximately $2765 per death for heart disease, $2724 for stroke, and $639 for VTE.

## 4 | DISCUSSION

To our knowledge, this is the first comprehensive analysis of NIH funding for VTE research. After adjusting for inflation, NIH support for VTE research increased by approximately 60% over the past decade. However, despite the substantial disease burden, our results suggest that overall investment remains relatively low compared with that for other leading causes of vascular mortality.

In 2023, we found that the NIH invested $2765 per annual death for heart disease, $2724 for stroke, and only $639 for VTE. The mortality figures for heart disease and stroke were derived from CDC National Vital Statistics [1], whereas VTE mortality estimates were sourced from separate CDC documentation [2]. Notably, the NIH Categorical Spending Report has recently added mortality estimates for select disease categories, such as heart disease and stroke [5], which differ from the CDC-based figures used in our analysis. For example, the NIH reported 2023 mortality estimates of 1 366 014 for heart disease and 222 212 for stroke [5]. Using these values, the per-death investment estimates would change to $1378 for heart disease and $1994 for stroke, both still significantly higher than the $639 per death invested in VTE research.

Beyond the monetary comparisons, these findings offer several critical insights into the landscape of federally funded VTE research and the value of data in NIH RePORTER. For example, the number of annual mentored K awards, which serve as an indicator of the early-career investigator pipeline, increased by more than 300% during the study period. Additionally, these data provide researchers in the field with access to commonly funded grant mechanisms, supporting institutions, and potential mentors or collaborators.

This analysis is timely given the proposed contraction of funds supporting the NIH portfolio [13] and the potential restructuring of scientific institutes [14], including the National Heart, Lung, and Blood Institute (NHLBI), the primary funder of VTE research. Transparent and accessible reporting of federally funded VTE research is essential to inform future advocacy efforts. While the inclusion of VTE in the NIH’s annual Categorical Spending Report is warranted, in its absence, the LLM used in this analysis offers an efficient alternative for accessing funding data without the burden of manual review. Although not without limitations, this LLM has demonstrated strong performance compared with manual review in other unreported disease categories [15] and in the current study, with a kappa statistic of 0.71.

Several additional limitations of this analysis are worth noting. First, the search strategy and inclusion/exclusion criteria were developed by the study authors and intentionally designed to be liberal to capture all research on VTE. Modifications to either of these methodological steps could impact the resulting estimates and should be explored in future studies. Second, interrater reliability between the LLM and human reviewers was assessed for a single funding year only. Cohen’s kappa between the 2 human reviewers was higher than Fleiss’ kappa incorporating the LLM, but large-scale human review is impractical due to the significant burden of manual review. Third, this analysis did not account for federal grants funded outside the NIH, such as those from the Food and Drug Administration, the Department of Veterans Affairs, or the Agency for Healthcare Research and Quality. Fourth, mortality was used as the comparator metric for funding, which provides an incomplete assessment of disease burden. Future analyses should consider incorporating metrics that also account for morbidity, such as disability-adjusted life years. Fifth, the annual number of VTE-related events in the U.S. is uncertain. We used a CDC estimate of 100 000 deaths per year to calculate the research investment per death, although this figure may underestimate the true burden and therefore lead to an even lower investment than reported. Also, funding for heart disease and stroke was based on published NIH estimates derived from their internal automated indexing tool [16]. Finally, the data used in this analysis do not offer insights into the underlying causes of the potential funding disparities identified. Specifically, the total number of grant applications submitted for each disease category is unknown, and VTE research funding may reflect a smaller pool of investigators submitting competitive grant proposals.

## 5 | CONCLUSION

NIH investment in VTE research has increased over the past decade, but remains low compared with other leading causes of vascular mortality in the U.S. The reasons for this funding disparity are unclear; however, these findings may help guide future advocacy efforts and inform federal funding priorities. Additionally, in the absence of VTE reporting in the NIH Categorical Spending Report, LLMs can provide accessible estimates of the annual federal investment in VTE research.

## Supplementary Material

1

The online version contains supplementary material available at https://doi.org/10.1016/j.jtha.2026.01.020.

## Figures and Tables

**TABLE 1:** Inclusion and exclusion criteria for venous thromboembolism grant categorization.

**Inclusion criteria:** any one or more of the following:
Studies designed to improve the prevention, diagnosis, treatment, or outcomes of VTE, deep vein thrombosis, or pulmonary embolism. Use of a VTE, pulmonary embolism, or deep vein thrombosis preclinical model to study thrombogenesis or clot propagation. Investigation of VTE, pulmonary embolism, or deep vein thrombosis risk factors for the prediction, prevention, or management of VTE, pulmonary embolism, or deep vein thrombosis. Studies investigating the epidemiology or public health impact of VTE, pulmonary embolism, or deep vein thrombosis. Research on anticoagulant therapies (eg, direct oral anticoagulants, heparins, and thrombolytics) aimed at the prevention or treatment of VTE, pulmonary embolism, or deep vein thrombosis. Studies aimed at procedural treatment for VTE, pulmonary embolism, or deep vein thrombosis (eg, mechanical thrombectomy/em-bolectomy and catheter-directed thrombolysis) Studies evaluating VTE, pulmonary embolism, or deep vein thrombosis prophylaxis (eg, compression devices and IVC filters). Research on biomarkers, imaging modalities, or scoring systems for VTE, pulmonary embolism, or deep vein thrombosis diagnosis, treatment, or risk stratification. Study of complications of VTE, including postthrombotic syndrome, chronic thromboembolic pulmonary hypertension, etc. Funding for research centers, networks, or collaborative groups with a stated focus on VTE, pulmonary embolism, or deep vein thrombosis.
**Exclusion criteria:** any one or more of the following:
Studies focused on arterial thrombosis, superficial vein thrombosis, cerebral venous sinus thrombosis, splanchnic vein thrombosis, or other nonpulmonary embolism or nondeep vein thrombosis locations. Research on atrial fibrillation-related thromboembolism, unless there is a direct aim related to VTE. Studies on hematologic disorders, unless directly aimed at VTE, pulmonary embolism, or deep vein thrombosis pathophysiology or management. Research on surgical or interventional procedures (eg, cardiovascular surgeries and endovascular procedures), unless VTE, pulmonary embolism, or deep vein thrombosis prevention or treatment is a primary aim. Studies of general inflammation, endothelial dysfunction, or coagulation, unless explicitly focused on VTE. Studies with aims unrelated to VTE, pulmonary embolism, or deep vein thrombosis.

IVC, inferior vena cava; VTE, venous thromboembolism.

**TABLE 2:** Larger language model parameters used.

Parameter	Description	Value
Model name	Full name of the model used	GPT-4o mini
Date accessed	Date when the model was used	May 9, 2025
Model URL/repository	URL to the model’s API endpoint, web interface location, or repository	Microsoft Azure Custom Endpoint
Model version	Specific version or variant of the model	July 18, 2024
Personalization: memory	Can the model access context from previous conversations during inference/prediction	No
Personalization: temperature	Parameter that tunes the consistency of the results (0: most consistent; 2: least consistent)	0
Custom functions/tools	List of custom functions/tools used by the model	LabeLMaker (release candidate 1) https://github.com/UABPeriopAI/label_maker

API, application programming interface.

**TABLE 3:** Descriptive summary of National Institutes of Health-funded venous thromboembolism grants from 2015 to 2024.

Funding year	2015	2016	2017	2018	2019	2020	2021	2022	2023	2024
Grants, *N*	78	75	76	104	112	129	137	130	135	138
Principal investigators, *N*	69	67	69	96	104	114	123	116	126	127
Grant type										
Newly funded	17 (21.8)	18 (24.0)	20 (2.63)	28 (26.9)	26 (23.2)	31 (24.0)	42 (30.7)	29 (22.3)	25 (18.5)	24 (17.4)
Continuing	61 (78.2)	57 (76.0)	56 (73.7)	76 (73.1)	86 (76.8)	98 (76.0)	95 (69.3)	101 (77.7)	110 (81.5)	114 (82.6)
Mechanism										
R01	30 (38.5)	32 (42.7)	32 (42.1)	41 (39.4)	41 (36.6)	49 (38.0)	60 (43.8)	59 (45.4)	55 (40.7)	61 (44.2)
R series (other than R01)	9 (11.5)	11 (14.7)	12 (15.8)	16 (15.4)	21 (18.8)	22 (17.1)	32 (23.4)	29 (22.3)	22 (16.3)	19 (13.8)
K award^a^	3 (3.8)	5 (6.7)	3 (3.9)	5 (4.8)	8 (7.1)	7 (5.4)	12 (8.8)	14 (10.8)	13 (9.6)	13 (9.4)
U series	13 (16.7)	13 (17.3)	6 (7.9)	8 (7.7)	8 (7.1)	3 (2.3)	4 (2.9)	5 (3.8)	4 (3.0)	2 (1.4)
Other^b^	23 (29.5)	14 (18.7)	23 (30.3)	34 (32.7)	34 (30.4)	48 (37.2)	29 (21.2)	23 (17.7)	41 (30.4)	43 (31.2)
Funding institute										
NHLBI	59 (75.6)	59 (78.7)	53 (69.7)	73 (70.2)	73 (65.2)	75 (58.1)	95 (69.3)	94 (72.3)	85 (63.0)	90 (65.2)
NICHD	10 (12.8)	5 (6.7)	11 (14.5)	17 (16.3)	21 (18.8)	31 (24.0)	14 (10.2)	5 (3.8)	25 (18.5)	27 (19.6)
Funding, $USD millions^c^										
All awards (total)	42.0	42.4	46.0	58.6	59.7	60.6	73.0	71.6	65.2	67.1
Median (IQR)	0.46 (0.26–0.60)	0.50 (0.28–0.65)	0.51 (0.24–0.83)	0.47 (0.26–0.79)	0.44 (0.21–0.72)	0.37 (0.15–0.69)	0.45 (0.20–0.75)	0.47 (0.20–0.71)	0.41 (0.17–0.67)	0.46 (0.16–0.68)
K Awards^a^ (total)	0.62	1.1	0.66	1.0	1.6	1.4	2.1	2.3	2.3	2.3

Data are *n* (%) unless otherwise specified.

NHLBI, National, Heart, Lung, and Blood Institute; NICHD, National Institute of Child Health and Human Development.

a Defined as K01, K08, K23, and K99 mechanisms.

b ZIA, DP5, F series, P series, SC1, SC3, and T32.

c Adjusted for inflation.

## Data Availability

The analysis used publicly available grant information and thus did not require institutional review board approval. The fully categorized dataset is provided in the Supplementary Table.
